# Serum Leptin Concentration Positively Correlates with Body Weight and Total Fat Mass in Postmenopausal Japanese Women with Osteoarthritis of the Knee

**DOI:** 10.1155/2011/580632

**Published:** 2011-01-24

**Authors:** Jun Iwamoto, Tsuyoshi Takeda, Yoshihiro Sato, Hideo Matsumoto

**Affiliations:** ^1^Institute for Integrated Sports Medicine, Keio University School of Medicine, 35 Shinanomachi, Shinjuku-ku, Tokyo 160-8582, Japan; ^2^Department of Neurology, Mitate Hospital, Tagawa, Fukuoka 826-0041, Japan

## Abstract

The objective of the present study was to identify factors correlated with the serum leptin concentration in women with knee OA. Fifty postmenopausal Japanese women with knee OA (age: 50–88 years) were recruited in our outpatient clinic. Plain radiographs of the knee were taken, and urine and blood samples were collected. Dual-energy X-ray absorptiometry (DXA) scanning was performed for the whole body and lumbar spine, and factors correlated with the serum leptin concentration were identified. A simple linear regression analysis showed that body weight, body mass index, whole-body bone mineral density (BMD), total fat mass, and total fat percentage, but not age, height, lumbar spine BMD, lean body mass, serum and urinary bone turnover markers, or the radiographic grade of knee OA, were significantly correlated with the serum leptin concentration. A multiple regression analysis showed that among these factors, only body weight and total fat mass exhibited a significant positive correlation with the serum leptin concentration. These results suggest that the serum leptin concentration might be related to increases in body weight and total fat mass, but not to BMD or bone turnover markers, in postmenopausal women with OA.

## 1. Introduction

Leptin, the product of the *ob* gene, is a peptide hormone secreted primarily by the adipocytes and plays an important role in the regulation of body weight by centrally inhibiting food intake and stimulating energy expenditure [1]. Leptin enters the circulation and crosses the blood-brain barrier to reach its primary target, receptors in the hypothalamus. However, a clinical study has shown that the serum leptin concentration is elevated in obese persons and is correlated with the percentage of body fat [2]. Thus, obesity is more likely to be caused by central mechanisms regulating food intake and energy expenditure than by defective signaling originating in adipocytes and affecting these central mechanisms [2].

Leptin regulates bone metabolism [3]. Leptin decreases bone formation and increases bone resorption via the sympathetic nervous system [4, 5]. Conversely, circulating leptin also regulates bone metabolism directly by binding to leptin receptors on bone marrow stromal cells, osteoblasts, and osteoclasts, functioning to increase osteoblast activity and decrease osteoclast activity [6]. Clinically, the results of cross-sectional and longitudinal cohort studies examining relationships among the serum leptin concentration, bone mineral density (BMD), and/or bone formation and resorption markers remain contradictory [7–9]. A recent prospective study demonstrated that the initial serum leptin concentration, together with specific-body composition parameters, determined the loss in total and femoral neck BMD values in physically active older women [10].

Osteoarthritis (OA) of the knee is the most common type of arthritis and is the major cause of chronic musculoskeletal pain and mobility disability in elderly populations, representing a significant burden on health care provision. Well-established risk factors for OA include aging, obesity, and female sex [11]. The serum leptin concentration is known to be two to threefold higher in women than in men, independent of adiposity [12]. It has been hypothesized that leptin might be a systemic or local factor mediating the metabolic link between obesity and OA and partially accounting for the gender disparity of this disease [13]. 

Accordingly, leptin was speculated to mediate the metabolic link connecting fat mass, knee OA, and BMD in women. However, very few studies have addressed this issue in women with knee OA. The objective of the present study was to identify possible correlations between the serum leptin concentration and various factors including age, height, body weight, body mass index (BMI), BMD, lean body mass, total fat mass, total fat percentage, bone turnover markers, and the radiographic grade of OA in women with knee OA. 

## 2. Subjects and Methods

### 2.1. Subjects

Postmenopausal Japanese women with OA of the knee who visited our outpatient clinic (orthopaedics and sports medicine clinic) in 2002 or 2003 were recruited. All the patients had consulted experts in knee OA treatment at our clinic because of knee pain. The diagnosis of OA of the knee was made based on the clinical symptoms, the results of a physical examination, and X-ray findings of the knee. All the patients had mild, moderate, or severe OA of the knee (grades 1–4) according to the Kellgren and Lawrence method of grading (grade 0, normal; grade 1, possible osteophytes only; grade 2, definite osteophytes and possible joint-space narrowing; grade 3, moderate osteophytes and/or definite joint-space narrowing; and grade 4, large osteophytes, severe joint-space narrowing, and/or bony sclerosis) [14]. Because pharmacological treatments such as oral nonsteroidal anti-inflammatory drugs (NSAIDs) and intra-articular injections of hyaluronates are effective for symptom relief [15, 16], most of the patients were receiving weekly to monthly intraarticular injections of hyaluronate sodium and/or oral NSAIDs.

After one-month of the cessation of intraarticular injections of hyaluronate sodium, dual-energy X-ray absorptiometry (DXA) scanning was performed on the whole body and the whole lumbar spine. Urine and serum samples were collected from all the patients between 9:00 am and 11:00 am, and the serum leptin and urinary and serum bone turnover markers were measured. Factors correlated with the serum leptin level were then determined. Informed consent was obtained from each patient. 

### 2.2. Measurements of Serum Leptin and Serum and Urinary Bone Turnover Markers

The urinary levels of pyridinoline and deoxypyridinoline were measured using high-performance liquid chromatography (HPLC) (normal range: 17.7–41.9 pmol/*μ*mol Cr and 2.8–7.6 nmol/mmol Cr, resp.) [17], and the urinary levels of cross-linked N-terminal telopeptide of type I collagen (NTX) were measured using an enzyme-linked immunosorbent assay (ELISA) (normal range: 9.3–54.3 nM BCE/mM Cr) [17]. The serum levels of bone-specific alkaline phosphatase (BAP) were measured using a chemiluminescent enzyme immunoassay (CLEIA) (normal range: 7.9–20.9 U/L) [17]. The serum levels of osteocalcin (OC) were measured using an immunoradiometric assay (IRMA) (normal range: 3.1–12.7 ng/mL). The serum levels of leptin were measured using a radioimmunoassay (RIA) (normal range: 2.5–21.8 ng/mL). 

### 2.3. DXA Scanning

First, DXA scanning was performed on the whole body in the supine position using a Norland XR-36 instrument (Norland, Fort Atkison, WI, USA). The BMD of the whole body, lean body mass, total fat mass, and total fat percentage were measured. Second, DXA scanning was also performed on the lumbar spine in the supine position, and the BMD of the lumbar spine (L2–L4) in the anteroposterior view was measured.

 According to the Japanese diagnostic criteria for the diagnosis of osteoporosis [18, 19], patients with a lumbar spine or hip BMD <70% of the young adult mean (YAM) or of 70–80% of the YAM and a history of osteoporotic fractures are diagnosed as having “osteoporosis”. Patients with a lumbar spine BMD, but not a whole-body BMD, ≧80% of the YAM and between 70%–80% of the YAM without any history of osteoporotic fractures are diagnosed as being “normal” and having “osteopenia”, respectively. Data regarding the YAM for whole-body BMD is not available. 

### 2.4. Statistical Analysis

A simple linear regression analysis was used to examine possible correlations between the serum leptin level and age; body weight; height; BMI; lumbar spine BMD; whole-body BMD; lean body mass; total fat mass; total fat percentage; serum and urinary bone turnover markers including serum BAP and osteocalcin and urinary pyridinoline, deoxypyridinoline, and NTX; and the radiographic grade of knee OA. A multiple regression analysis was used to determine factors correlated with the serum leptin level among the factors that were significantly correlated with the serum leptin level in a simple linear regression analysis. All statistical analyses were performed using the Stat View-J5.0 program on a Windows computer. A significance level of *P* < .05 was used for all the comparisons.

## 3. Results

### 3.1. Characteristics of Study Subjects

Table 1 shows the characteristics of the study subjects. The mean age was 66.4 years, and the mean BMI was 24.1 kg/m^2^ (normal range: 18.5–25.0 kg/cm^2^), corresponding to a “normal high” level for Japanese populations. The mean lumbar spine BMD was 0.864 g/cm^2^, which was 83.1% of the young adult mean (diagnosed as “normal BMD”). The mean radiographic grade of OA was 2.47. The mean serum leptin level was 9.7 ng/mL (within normal range). Although the mean urinary pyridinoline and deoxypyridinoline and the serum OC levels were within the normal ranges, the serum BAP and urinary NTX levels were higher than the normal ranges [17].

### 3.2. Correlations of Serum Leptin Level with Various Factors

**Figure 1:**
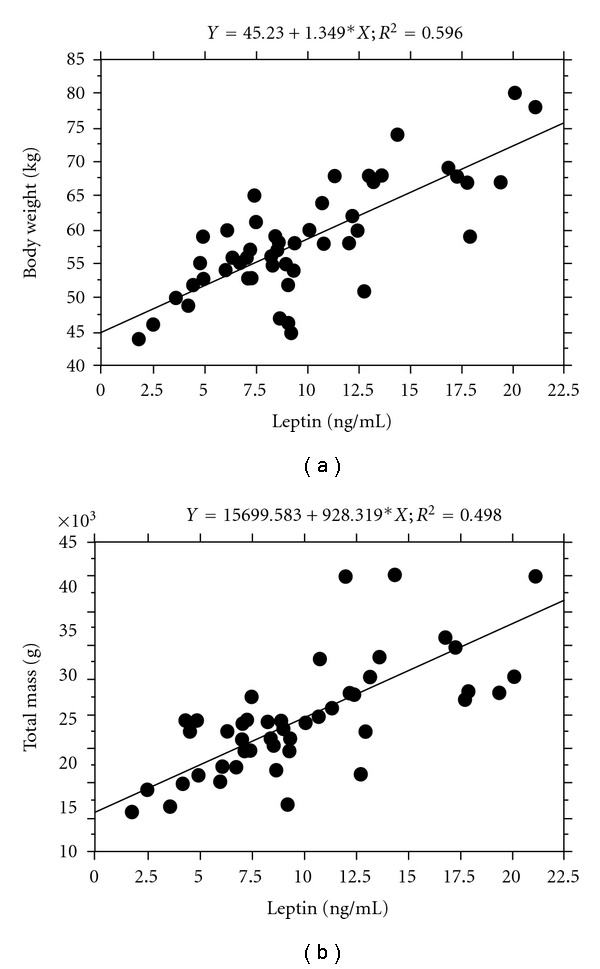
Correlations between the serum leptin level and body weight and total fat mass. A simple linear regression analysis was used to examine correlations of serum leptin level with body weight and total fat mass. Body weight and total fat mass had a significant positive correlation with serum leptin level (both *P* < .0001).

A simple linear regression analysis showed that five factors (body weight, BMI, whole-body BMD, total fat mass, and total fat percentage) but not age, height, lumbar spine BMD, lean body mass, serum and urinary bone turnover markers, or the radiographic grade of knee OA were significantly correlated with the serum leptin level (Table 2). A multiple regression analysis showed that among these five factors, body weight and total fat mass were significantly correlated with the serum leptin level (*r* ^2^ = 0.859, Table 3).  Figure 1 shows the correlations between the serum leptin level and the body weight and total fat mass according to simple linear regression analyses. 

## 4. Discussion

The present study confirmed that the serum leptin concentration was significantly correlated with the body weight and total fat mass in postmenopausal women with knee OA. These positive findings are consistent with the well-known fact that the serum leptin concentration is closely correlated with fat mass and decreases after weight loss [20]. OA is well known to be strongly correlated with a high BMI [20]. Gonzalez-Gay et al. [21] found a positive correlation between BMI and serum leptin in patients with severe rheumatoid arthritis receiving tumor necrosis factor-*α* (TNF-*α*) antagonist therapy. In the present study, a simple linear regression analysis showed a similar correlation in patients with knee OA.

The relationship between obesity and OA is an important public health issue. An experimental study showed that extreme obesity arising from impaired leptin signaling induced alterations in subchondral bone morphology without increasing the incidence of knee OA, suggesting that body fat, in and of itself, might not be a risk factor for joint degeneration [22]. Obesity alone does not cause knee OA, and leptin might be involved in OA because without leptin, obesity itself does not predispose an individual to OA [22]. Leptin receptors have been found in articular cartilage, further implying that leptin synthesis and secretion might play a role in OA [13]. Leptin might play a catabolic role in cartilage metabolism and may be a disadvantage factor involved in the pathological process of OA [23]. Thus, the high concentration of serum leptin in obese individuals might be associated with an increased risk of knee OA. As a result of the effects of sex hormones, the serum leptin concentration is higher in women than in men, even after adjustments for BMI, and this difference might be relevant to the influence of gender on the development and frequency of OA [24]. However, the present study did not show any significant correlation between the serum leptin concentration and the radiographic grade of knee OA. Thus, the elevated serum leptin concentrations might not have resulted in an increased risk of knee OA in our subjects.

Studies have shown that patients with knee OA might have a higher bone mineral content (BMC) and bone size, although the relation between knee OA and BMD remains to be established [25–34]. However, Weiss et al. [7] suggest that obese people might have a relatively low level of bone loss and a decreased risk of osteoporosis. The bone-sparing effects of body weight might possibly be caused by increased loading on the skeleton, the increased production of estrogen in adipose tissue, the increased bone formation caused by the anabolic effects of high levels of insulin, and/or high levels of bone-related hormones such as leptin [7]. The present study showed that postmenopausal women with knee OA (mean age: 66.4 years) had a normal lumbar spine BMD and a “normal high” BMI, despite the fact that women older than 65 years have an increased risk of osteoporosis [35]. The normal lumbar spine BMD might partly be attributable to the “normal high” BMI and the subsequent increases in loading to the skeleton. The significant correlation between the serum leptin concentration and whole-body BMD observed in the simple regression analysis disappeared in a multiple regression analysis, suggesting an interaction between body weight or total fat mass and whole-body BMD. The urinary NTX and serum BAP levels in our study subjects suggested that bone turnover was mildly increased, compared with in healthy premenopausal women [17], as a result of menopause, similar to previously reported postmenopausal women with osteoporosis [36]. The serum leptin concentration was not significantly correlated with the BMD and bone turnover markers, probably because it was not high enough to influence bone formation and resorption and, subsequently, the BMD.

Joint space narrowing, sclerosis of the subchondral bone, and the presence of osteophytes are typical structural features of knee OA. Thus, both articular cartilage and subchondral bone are considered to be involved in the pathogenesis of knee OA. However, recent evidence has shown that increased local bone turnover, decreased BMC and stiffness, and trabecular bone loss have been observed in the subchondral bone structure of knee OA [37–39]; therefore, subchondral bone abnormalities might be a major factor in disease progression. The Framingham Study showed that a high BMD and BMD gain decreased the risk of radiographic knee OA progression [40]. Thus, the subchondral bone mass and structure and the whole-body BMD, if it influences the subchondral bone, might be associated with the progression of knee OA. In the present study, however, no significant association was found between the radiographic grade of knee OA and the lumbar spine and whole-body BMD (data not shown), probably because of the small sample sizes in the subanalyses.

Weight loss is correlated with a decrease in the progression of OA, and the serum leptin concentration decreases after weight loss [20]. Thus, studying the effect of interventions aimed at weight loss on the progression of knee OA, serum leptin concentration, and BMD in patients with knee OA would be interesting. Adiponectin, an adipose-modulated biochemical signal, might play an important role in the maintenance of the total BMC and regional BMD in physically active older women [10]. Thus, examining the correlations among serum adiponectin concentration, the radiographic grade of knee OA, BMD, body size, and fat mass in women would also be interesting. Further research is needed to clarify these issues. 

In conclusion, the present study evaluated the serum leptin concentration, the radiographic grade of knee OA, BMD, body size, and fat mass in postmenopausal women with knee OA. The serum leptin concentration and the lumbar spine BMD were normal. However, the BMI was “normal high,” probably because of a mild impairment in the central mechanisms of leptin signaling. The present study revealed that body weight and total fat mass, but not the BMD or the radiographic grade of OA, exhibited a significant positive correlation with the serum leptin concentration in postmenopausal women with knee OA. 

## Figures and Tables

**Table 1:** Characteristics of study subjects.

	Mean ± SD	Range
Age (years)	66.4 ± 8.2	50–88
Height (m)	1.56 ± 0.05	1.47–1.65
Body weight (kg)	58.4 ± 8.2	44–80
Body mass index (kg/m^2^)	24.1 ± 3.4	16.9–33.3
Lumbar spine BMD (g/cm^2^)	0.864 ± 0.204	0.524–1.316
Whole body BMD (g/cm^2^)	0.866 ± 0.108	0.647–1.109
Lean body mass (g)	34229 ± 7548	20720–64203
Total fat mass (g)	24734 ± 6135	14302–41201
Total fat percent (%)	40.8 ± 5.3	29.7–51.5
Serum BAP (U/L)	28.1 ± 9.8	11.7–49.8
Serum OC (ng/mL)	5.7 ± 2.2	1.7–11.9
Urinary pyridinoline (pmol/*μ*mol Cr)	32.5 ± 9.3	19.3–63.6
Urinary deoxypyridinoline (nmol/mmol Cr)	6.5 ± 2.0	2.8–10.1
Urinary NTX (nmol BCE/mmol Cr)	57.9 ± 21.4	8.2–95.7
Serum leptin (ng/mL)	9.7 ± 4.7	1.8–21.1
Radiographic grade	2.47 ± 1.00	1–4

BMD: bone mineral density, BAP: bone-specific alkaline phosphatase, OC: osteocalcin, NTX: cross-linked N-terminal telopeptides of type I collagen, BCE: bone collagen equivalent, Cr: creatinine.

Normal ranges of urinary pyridinoline, deoxypyridinoline, and NTX were 17.7–41.9 pmol/*μ*mol Cr and 2.8–7.6 nmol/mmol Cr, and 9.3–54.3 nM BCE/mM Cr, respectively. Normal ranges of serum BAP, OC, and leptin were 7.9–20.9 U/L, 3.1–12.7 ng/mL, and 2.5–21.8 ng/mL, respectively.

**Table 2:** Correlations of serum leptin level with various factors by simple regression analysis.

	Correlation coefficient	*P* value
Age (years)	−0.240	NS
Height (m)	0.162	NS
Body weight (kg)	0.792	<.0001
Body mass index (kg/m^2^)	0.693	<.0001
Lumbar spine BMD (g/cm^2^)	0.255	NS
Whole body BMD (g/cm^2^)	0.440	.0014
Lean body mass (g)	0.189	NS
Total fat mass (g)	0.706	<.0001
Total fat percent (%)	0.511	<.0001
Serum BAP (U/L)	−0.085	NS
Serum bone Gla protein (ng/mL)	−0.267	NS
Urinary pyridinoline (pmol/*μ*mol Cr)	0.138	NS
Urinary deoxypyridinoline (nmol/mmol Cr)	0.086	NS
Urinary NTX (nmol BCE/mmol Cr)	−0.151	NS
Radiographic grade	−0.110	NS

A simple linear regression analysis was used to examine correlations of serum leptin level with various factors. BMD: bone mineral density, BAP: bone-specific alkaline phosphatase, NTX: cross-linked N-terminal telopeptides of type I collagen, BCE: bone collagen equivalent, Cr: creatinine, NS: not significant.

**Table 3:** Correlations of serum leptin level with five factors by multiple regression analysis.

	Regression coefficient	Standard error	*P* value
Body weight (kg)	0.331	0.123	.0098
Body mass index (kg/m^2^)	−0.119	0.283	NS
Whole body BMD (g/cm^2^)	1.776	4.926	NS
Total fat mass (g)	3.145*E* − 4	1.943*E* − 4	.0275
Total fat percent (%)	−0.087	0.178	NS

A multiple regression analysis was used to examine correlations of serum leptin level with five factors that had a significant correlation by a simple regression analysis. BMD: bone mineral density, NS: not significant.
